# Supplementary material for: LARP3, LARP7, and MePCE are involved in the early stage of human telomerase RNA biogenesis
Source: Nat Commun. 2024 Jul 15;15:5955. doi: 10.1038/s41467-024-50422-w (PMC11250828; doi:10.1038/s41467-024-50422-w)
Supplement: Supplementary file 1 — Supplementary Information [file 41467_2024_50422_MOESM1_ESM.pdf]

Supplementary information

Supplementary Table 1. **Primers used to generate DNA templates for *in vitro* transcription.**

**Name**	**Sequence**	**Description**
CKOligo-64	TACGATTTAGGTGACACTATAGGGTTGC GGAGGGTGGGCCTGG	FW primer for hTR (+1)
CKOligo-70	GGTCCCCGGGAGGGGCG	RV primer for hTR (+196~+212)
CKOligo-71	ACTCGCTCCGTTCCTCTTCCTG	RV primer for hTR (+367~+388)
CKOligo-72	GCATGTGTGAGCCGAGTCCTGG	RV primer for hTR (+430~+451)
CKOligo-85	AAAGCGAACTGCATGTGTGAGCCGA	RV primer for hTR (+437~+461)
CKOligo-86	TTTTTAAAGCGAACTGCATGTGTGAGC CGA	RV primer for hTR (+437~+461) +5a
CKOligo-79	AACTGCATGTGTGAGCCGAGTCCT	RV primer for hTR (+432~+455)
CKOligo-80	TTTTTAACTGCATGTGTGAGCCGAGTCC T	RV primer for hTR (+432~+455) +5a
CKOligo-96	TTTTTAGAGCGAACTGCATGTGTGAGC CGA	RV primer for hTR (+437~+461) +5a (U460C)
CKOligo-226	TAATACGACTCACTATAGGGGACCTGC GGCGGGTCGC	FW primer for hTR (+206~+225)

Supplementary Table 2. **Plasmids used in this study.**

**Plasmid name**	**Description**
pMG80	WT hTR in pACGFP1-1
pTL07	GG375/6AT hTR in pACGFP1-1
pTL10	375-377GGAdel hTR in pACGFP1-1
pCMV3-LARP3-HA	LARP3 overexpression

Supplementary Table 3. **Antibodies used for Western blotting in this study.**

Target	Source	Cat#/reference	Dilution	Application
DHX36	Bethyl Laboratories	A300-525A	1:5000	WB
DKC1	Bethyl Laboratories	A302-591A	1:5000	WB
DKC1	SANTA CRUZ	sc-373956		IP
NHP2	Proteintech Group	15128-1-AP	1:1000	WB
NOP10	ABclonal	A18250	1:2000	WB
NAF1	Bethyl Laboratories	A303-911A	1:1000	WB
GAR1	Proteintech Group	11711-1-AP	1:1000	WB
TCAB1 (WRAP53)	Proteintech Group	14761-1-AP	1:1000	WB
LARP3	Bethyl Laboratories	A303-901A	1:15000	WB/IP
LARP3	Bethyl Laboratories	A303-902A	1:15000	WB
LARP7	Bethyl Laboratories	A303-723A	1:10000	WB/IF
MePCE	Bethyl Laboratories	A304-184A	1:5000	WB/IF
PARN	Bethyl Laboratories	A303-561A	1:10000	WB
LAMIN A/C	ABclonal	A19524	1:10000	WB
GAPDH	SANTA CRUZ	sc-32233	1:10000	WB
TUBULIN	ABclonal	AC012	1:5000	WB
COILIN	abcam	ab11822	0.5 μg/ml	IF
Normal Mouse IgG	Croyez	C06003		IP
Goat anti-rabbit IgG (H+L)-HRP	Croyez	C04003	1:5000	WB
Goat anti-mouse IgG (H+L)-HRP	Croyez	C04001	1:5000	WB
Anti-mouse IgG-heavy and light chain cross-adsorbed antibody	Bethyl Laboratories	A90-516D6	1:5000	WB
FITC-conjugated AffiniPure Goat Anti-Mouse IgG (H+L)	Jackson ImmunoResearch	115-095-003	1:200	IF
VeriBlot for IP detection reagent (HRP)	abcam	AB131366	1:1000	WB

Supplementary Table 4. **shRNA plasmids used in this study.**

Target	Plasmid	Target Sequence (5′ to 3′)
Luciferase	pLKO.1-Luciferase	GCGGTTGCCAAGAGGTTCCAT
LARP3	pLKO.1-LARP3-1	GCTGAAATGAAATCTCTAGAA
LARP3	pLKO.1-LARP3-2	CCTGCATCCAAACAACAGAAA
LARP3	pLKO.1-LARP3-3	CCAAGGCAGAACTCATGGAAA
LARP7	pLKO.1-LARP7-1	CTTGAGCTGTTCTTGGGAGAT
MePCE	pLKO-TRC005-MePCE-1	GAACTACTACCGAATCCAATT
PARN	pLKO.1-PARN-2	CCCAGACTCTTGGATACTAAA

Supplementary Table 5. **Primers used for northern blotting in this study.**

<table>
<tr><th>Name</th><th>Sequence</th><th colspan="2">Target</th></tr>
<tr><td>CKOligo-1</td><td>GGGTTGCGGAGGGTGGGC</td><td>FW primer for hTR (+1~+18)</td><td rowspan="2">hTR</td></tr>
<tr><td>CKOligo-2</td><td>CCGACTTTGGAGGTGCCTTC</td><td>RV primer for hTR (+580~+599)</td></tr>
<tr><td>CKOligo-64</td><td>TACGATTTAGGTGACACTAT AGGGTTGCGGAGGGTGGGC CTGG</td><td>FW primer for hTR with SP6 promoter (+1~+22)</td><td rowspan="2">hTR 5' end</td></tr>
<tr><td>CKOligo-70</td><td>GGTCCCCGGGAGGGGCG</td><td>RV primer for hTR (+196~+212)</td></tr>
<tr><td>CKOligo-72</td><td>GCATGTGTGAGCCGAGTCC TGG</td><td>RV primer for hTR (+430~+451)</td><td>hTR 3' end</td></tr>
<tr><td>CKOligo-7</td><td>GCGAACGCAGTCCCCCACT ACCACAAATTA</td><td>human U1 Northern probe</td><td>U1 snRNA</td></tr>
</table>

Supplementary Table 6. **Primers used for qRT–PCR.**

<table>
<tr><th>Name</th><th>Sequence</th><th>Target</th></tr>
<tr><td>CKOligo-14</td><td>FW: GCGAAGAGTTGGGCTCTGTCA</td><td rowspan="2">Total hTR</td></tr>
<tr><td>CKOligo-15</td><td>RV: TTCCTCTTCCTGCGGCCTGAAA</td></tr>
<tr><td>CKOligo-16</td><td>FW: CTTTCAGGCCGCAGGAAGAGGAA</td><td rowspan="2">Extended hTR</td></tr>
<tr><td>CKOligo-17</td><td>RV: GGTGACGGATGCGCACGAT</td></tr>
<tr><td>CKOligo-18</td><td>FW: TGCTGAGGATTTGGAAAGGG</td><td rowspan="2">HPRT</td></tr>
<tr><td>CKOligo-19</td><td>RV: ACAGAGGGCTACAATGTGATG</td></tr>
<tr><td>CKOligo-20</td><td>FW: GATCCTCTAGACTCCACCTCTC</td><td rowspan="2">ATP5β</td></tr>
<tr><td>CKOligo-21</td><td>RV: AGAAAGTTCATCCATACCCAGG</td></tr>
<tr><td>CKOligo-22</td><td>FW: ACATCGCTCAGACACCATG</td><td rowspan="2">GAPDH</td></tr>
<tr><td>CKOligo-23</td><td>RV: TGTAGTTGAGGTCAATGAAGGG</td></tr>
</table>

Supplementary Table 7. **Primers used for *in situ* hybridization.**

<table>
<tr><th>Name</th><th>Sequence</th><th>Target</th></tr>
<tr><td>CKFISH-1</td><td>Cy3-GCCCTTCTCAGTTAGGGTTA</td><td rowspan="7">hTR</td></tr>
<tr><td>CKFISH-2</td><td>Cy3-AAGTCAGCGAGAAAAACAGC</td></tr>
<tr><td>CKFISH-3</td><td>Cy3-TCTAGAATGAACGGTGGAAG</td></tr>
<tr><td>CKFISH-4</td><td>Cy3-CCAGCAGCTGACATTTTTTG</td></tr>
<tr><td>CKFISH-5</td><td>Cy3-GCTGACAGAGCCCAACTCTT</td></tr>
<tr><td>CKFISH-6</td><td>Cy3-GTCCCACAGCTCAGGGAATC</td></tr>
<tr><td>CKFISH-7</td><td>Cy3-CATGTGTGAGCCGAGTCCTG</td></tr>
</table>

Supplementary Table 8. **Primers used for hTR mutants with a site-directed mutagenesis kit.**

**Name**	**Sequence**	**Target**
CKOligo-531	CAGGCCGCAGGAAGAATAACGGAGC GAGTCCC	FW primer for GG375/6AT
CKOligo-532	GGGACTCGCTCCGTTATTCTTCCTGC GGCCTG	RV primer for GG375/6AT
CKOligo-585	CTCGCTCCGTTCTTCCTGCGGCCTGA AAG	FW primer for 375-377GGAdel
CKOligo-586	CTTTCAGGCCGCAGGAAGAACGGAG CGAG	RV primer for 375-377GGAdel

Supplementary Table 9. **Primers used for 3' RACE sequencing.**

**Name**	**Sequence**	**Target**
CKOligo-666	/5rApp/TTTAACCGCGAATTCCAG/3ddC/	3' linker
CKOligo-667	GACTGGAGTTCAGACGTGTGCTCTTC CGATCTCTGGAATTCGCGGTTAAA	Oligo annealing to 3' linker
CKOligo-668	CCTACACGACGCTCTTCCGATCTNNNN NNNNNNCAGGAAGAGGAACGGAGCG AGT	Oligo with barcode and downstream hTR template sequence
CKOligo-669	AATGATACGGCGACCACCGAGATCTAC ACTCTTTCCCTACACGACGCTCTTCCG ATC	PCR forward primer with Illumina P5 adaptor
CKOligo-676	CAAGCAGAAGACGGCATACGAGATGA TCTGGTGACTGGAGTTCAGACGTGTG	PCR reverse primer with Illumina P7 adaptor (sh-Luc)
CKOligo-685	CAAGCAGAAGACGGCATACGAGATGG ACGGGTGACTGGAGTTCAGACGTGTG	PCR reverse primer with Illumina P7 adaptor (sh-PARN)
CKOligo-686	CAAGCAGAAGACGGCATACGAGATGC GGACGTGACTGGAGTTCAGACGTGTG	PCR reverse primer with Illumina P7 adaptor (sh-LARP7)
CKOligo-687	CAAGCAGAAGACGGCATACGAGATTT TCACGTGACTGGAGTTCAGACGTGTG	PCR reverse primer with Illumina P7 adaptor (sh-MePCE)

Supplementary Figure 1

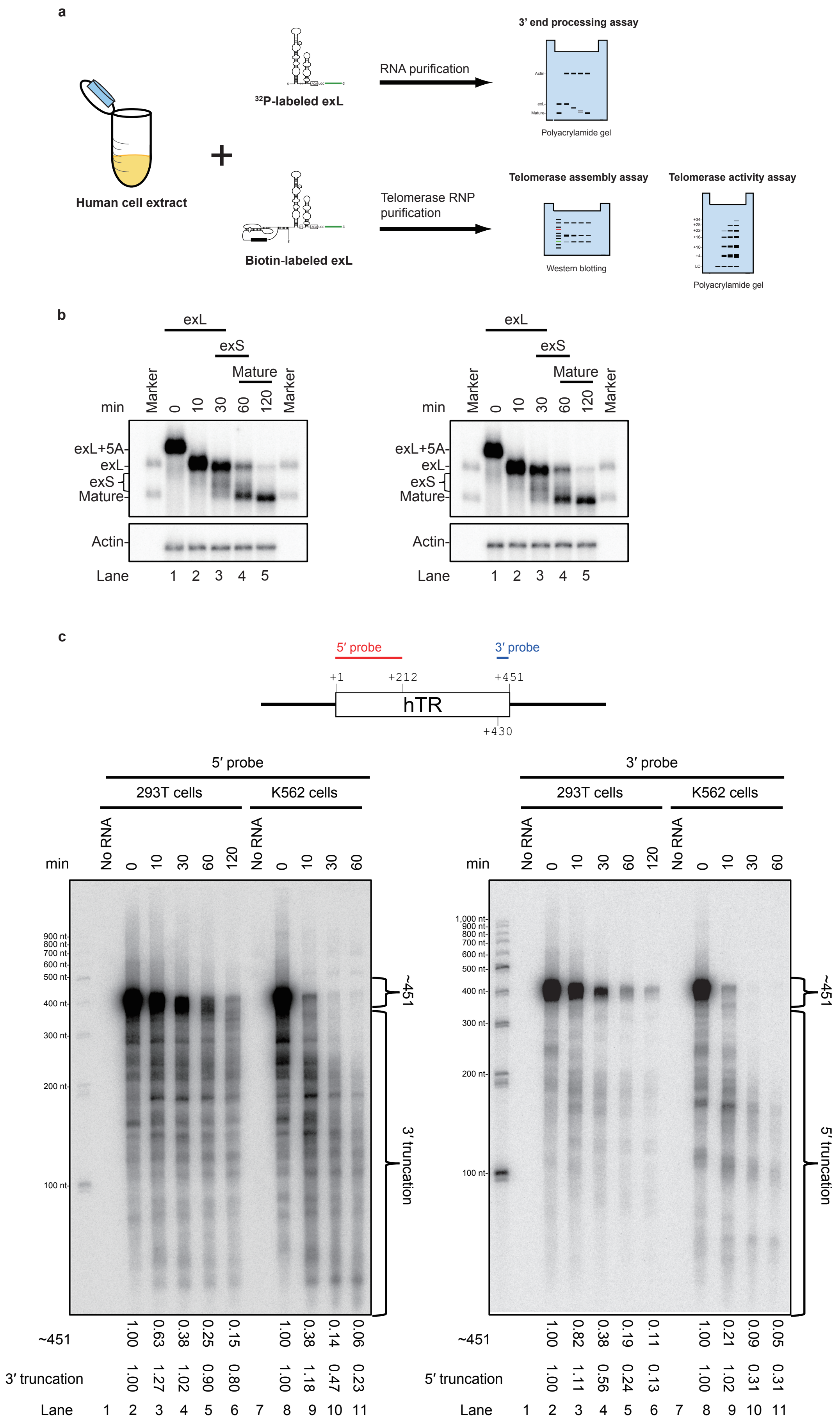

Supplementary Fig. 1 a Schematic showing human telomerase biogenesis and purification. **b** The in vitro 3′-end processing assay with ^{32}P-labelled hTR fragments (nucleotides from 206 to 461 with an oligo A tail) was carried out in 293T cell extracts at 37°C for the indicated times. RNA was purified and resolved on a 6% polyacrylamide gel containing 8 M urea. Actin was used as the loading control. **c** Northern blotting analysis of telomerase assembled on biotin-labelled hTR pulled down with streptavidin beads for the indicated times. Probes against the 5′ and 3′ regions of hTR were used.

Supplementary Figure 2

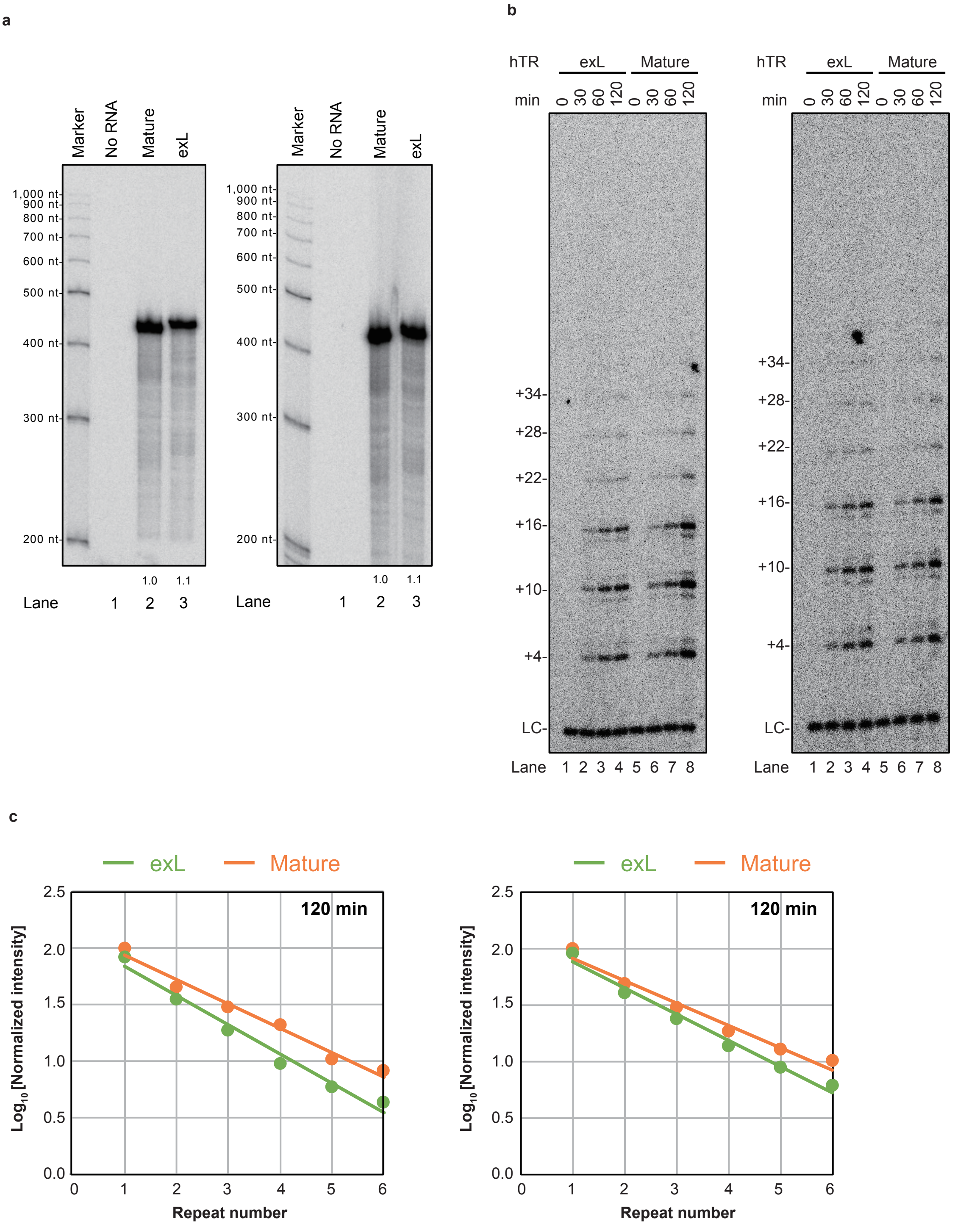

Supplementary Fig. 2 a Northern blotting analysis of the in vitro purified telomerase assembled on exL and the mature forms of hTR. **b** Telomerase activity of the in vitro-purified telomerase assembled on exL and the mature forms of hTR. **c** Telomerase processivity quantitation of in vitro-purified telomerase assembled on exL and mature forms of hTR. The intensity of each major band (+4, +10, +16, +22, +28, and so on) from the telomerase activity assay in b was quantified by phosphorimager analysis.

Supplementary Figure 3

a

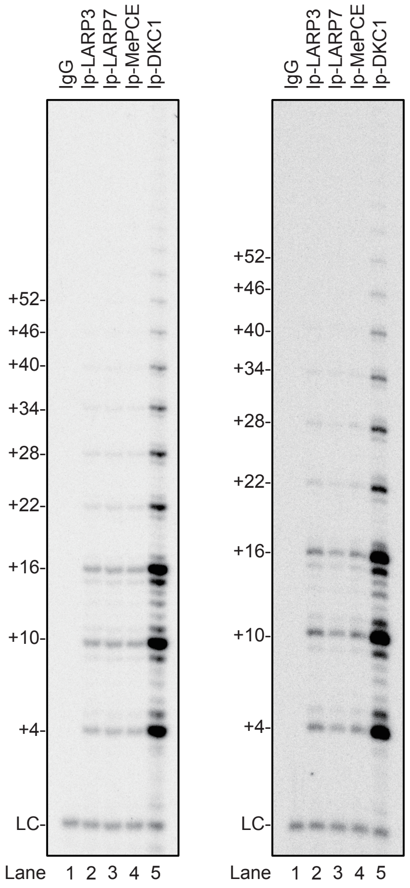

Supplementary Fig. 3 a Endogenous LARP3, LARP7, MePCE, and DKC1 were immunoprecipitated and subjected to a telomerase activity assay.

Supplementary Figure 4

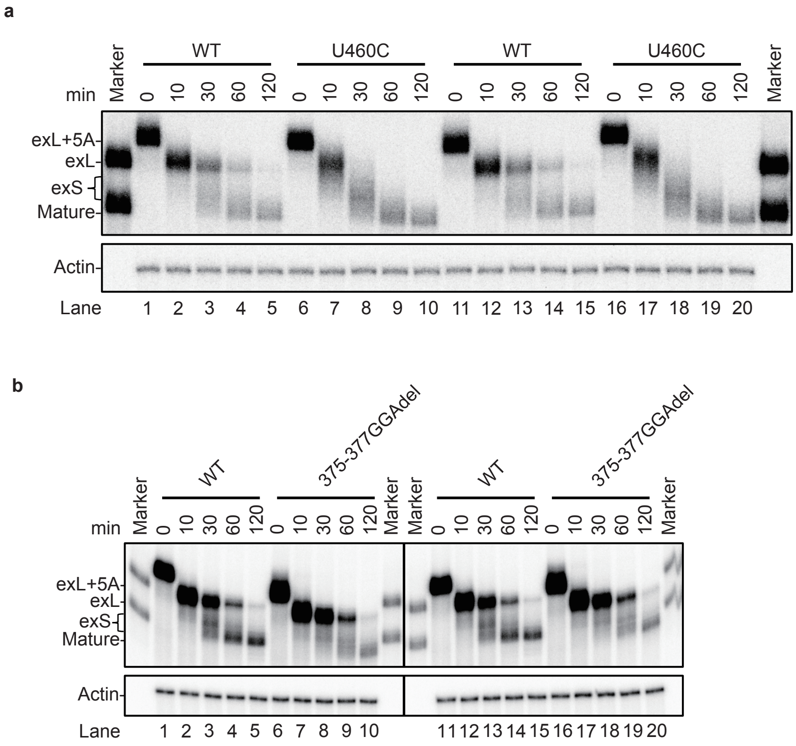

Supplementary Fig. 4 a and **b** The in vitro 3′-end processing assay with ^{32}P-labelled wild-type, U460C (a), or 375-377GGA-deleted (b) mutant hTR fragments (from nucleotides 206 to 461 with an oligo A tail) was carried out in 293T cell extracts at 37°C for the indicated times. RNA was purified and resolved on a 6% polyacrylamide gel containing 8 M urea. Actin was used as the loading control.

Supplementary Figure 5

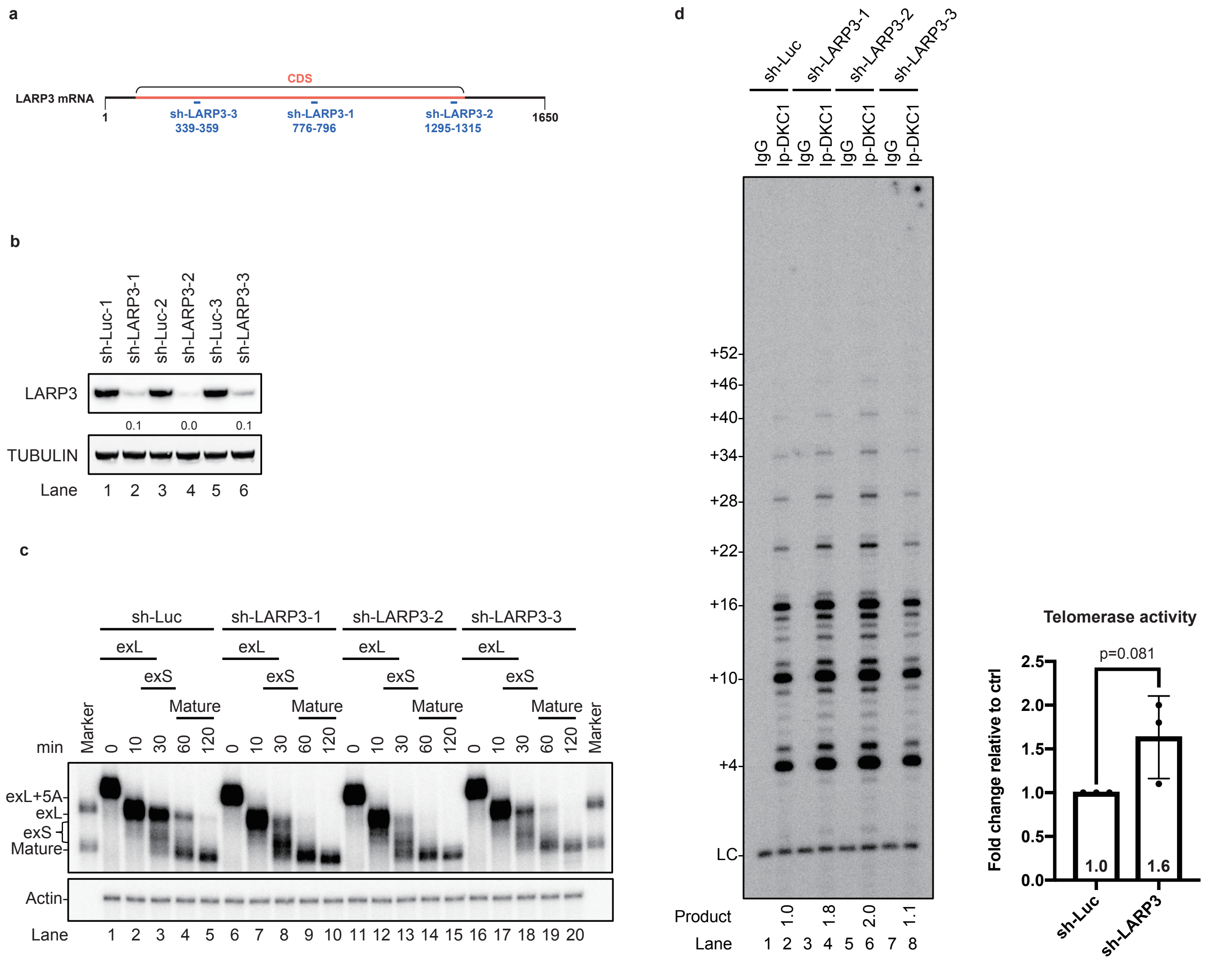

Supplementary Fig. 5 a Schematic showing the regions targeted by LARP3 shRNA. **b** Western blots of cell extracts prepared from 293T cells treated with three shRNAs targeting different regions of the LARP3 gene. Endogenous tubulin served as a loading control. **c** An in vitro 3′ end processing assay with ^{32}P-labelled hTR fragments (from nucleotides 206 to 461 with an oligo(A) tail) was carried out in cell extracts prepared from 293T cells treated with shRNAs targeting different regions of the LARP3 gene. **d** Endogenous DKC1 was immunoprecipitated from cell extracts prepared from 293T cells treated with three shRNAs targeting different regions of the LARP3 gene and subjected to a telomerase activity assay. The right panel of the bar graph shows that the mean values were calculated from experiments of three biological replicates, with the bars representing the SE. Dots represent data points from individual experiments. The significance of the change in telomerase activity between samples was calculated with a two-sided Student's t test; *$p < 0.05$, **$p < 0.01$, ***$p < 0.005$, ****$p < 0.001$.

Supplementary Figure 6

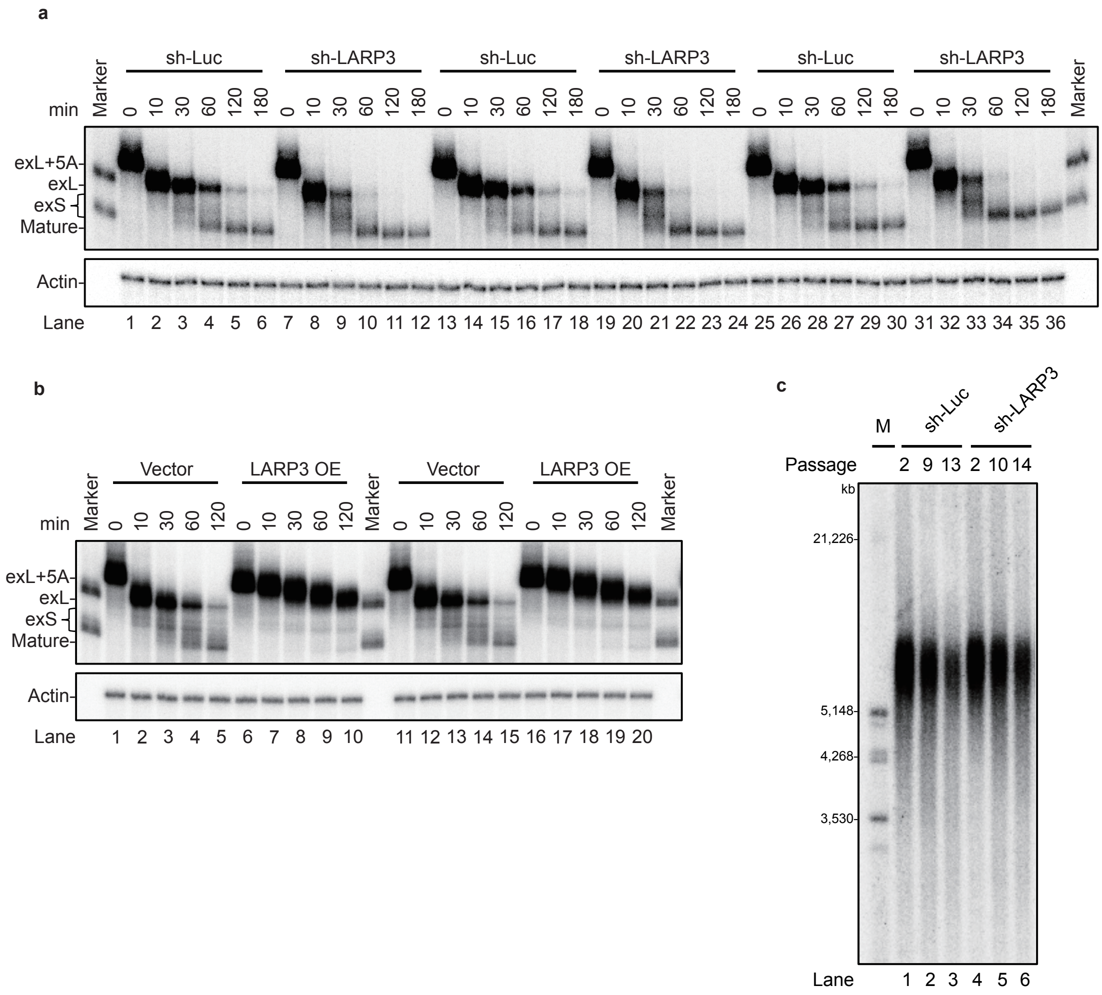

Supplementary Fig. 6 a and **b** The in vitro 3′-end processing assay with ^{32}P-labelled hTR fragments (from nucleotides 206 to 461 with an oligo A tail) was carried out with cell extracts prepared from 293T cells treated with either a shRNA targeting LARP3 (**a**) or transfected with an LARP3 plasmid (**b**) at 37°C for the indicated times. RNA was purified and resolved on a 6% polyacrylamide gel containing 8 M urea. Actin was used as the loading control. **c** Telomere lengths determined by TRF analysis of gDNA prepared from 293T cells treated with shRNAs targeting luciferase or LARP3.

Supplementary Figure 7

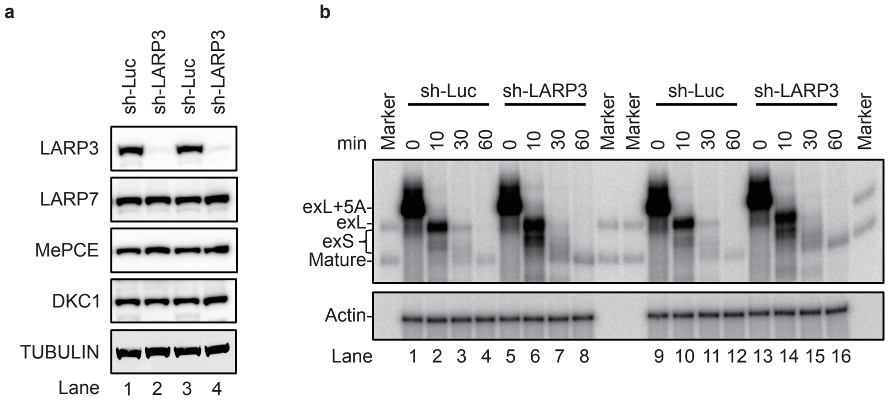

Supplementary Fig. 7 a Western blot analysis of extracts prepared from K562 cells treated with shRNA targeting luciferase or LARP3. **b** An in vitro 3′-end processing assay with ^{32}P-labelled hTR fragments (from nucleotides 206 to 461 with an oligo A tail) was carried out with the indicated cell extracts. RNA was purified and resolved on a 6% polyacrylamide gel containing 8 M urea. Actin was used as the loading control.

Supplementary Figure 8

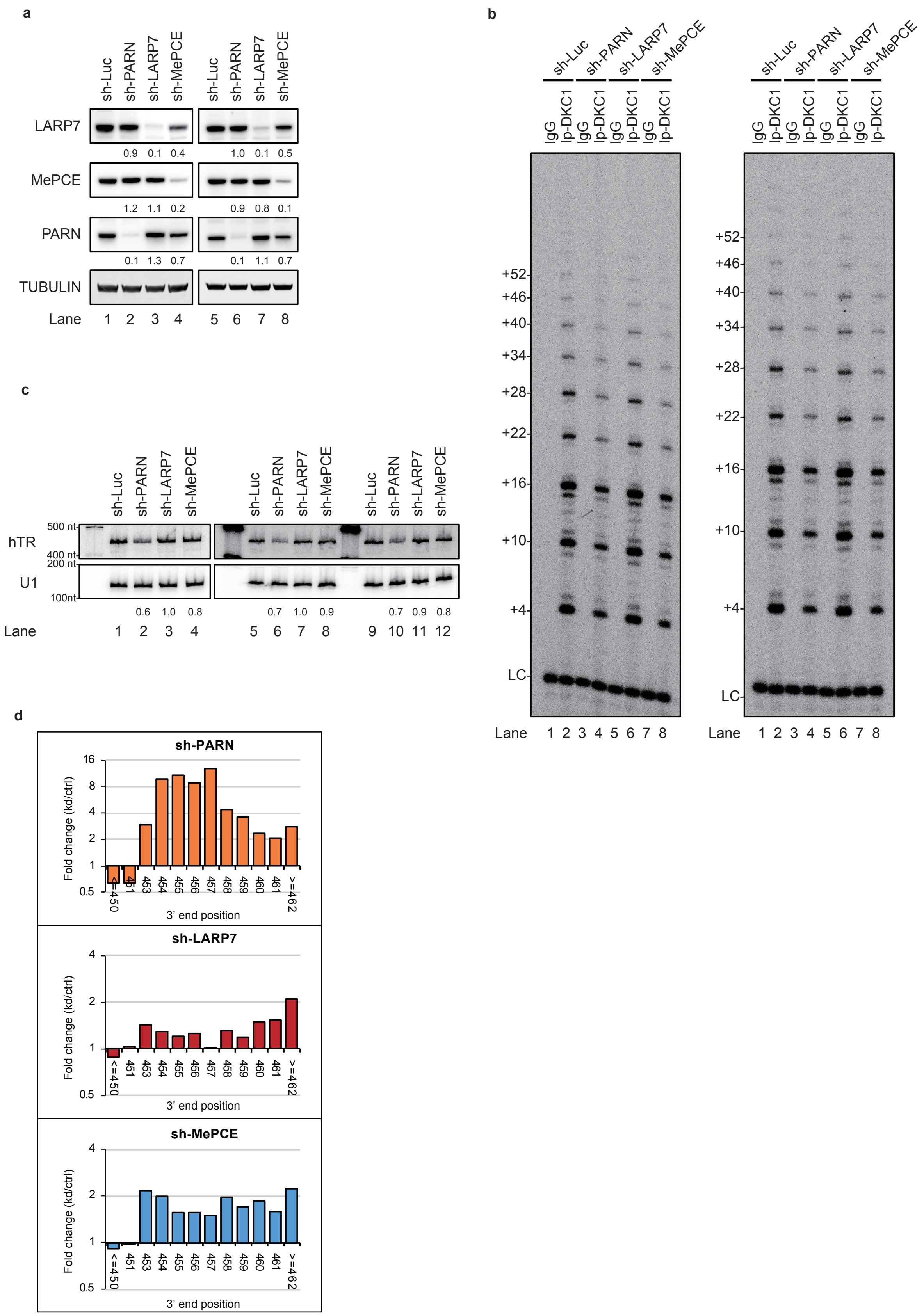

Supplementary Fig. 8 a Western blot analysis of extracts prepared from the HeLa cells treated with shRNAs targeting luciferase, PARN, LARP7, or MePCE. **b** Endogenous DKC1 was immunoprecipitated from cell extracts prepared from the HeLa cells treated with shRNAs targeting luciferase, PARN, LARP7, or MePCE and subjected to a telomerase activity assay. **c** Total RNA prepared from the HeLa cells treated with shRNAs targeting luciferase, PARN, LARP7, or MePCE was subjected to northern blotting. **d** Graphical representation of fold changes in distribution of hTR 3' end positions.

Supplementary Figure 9

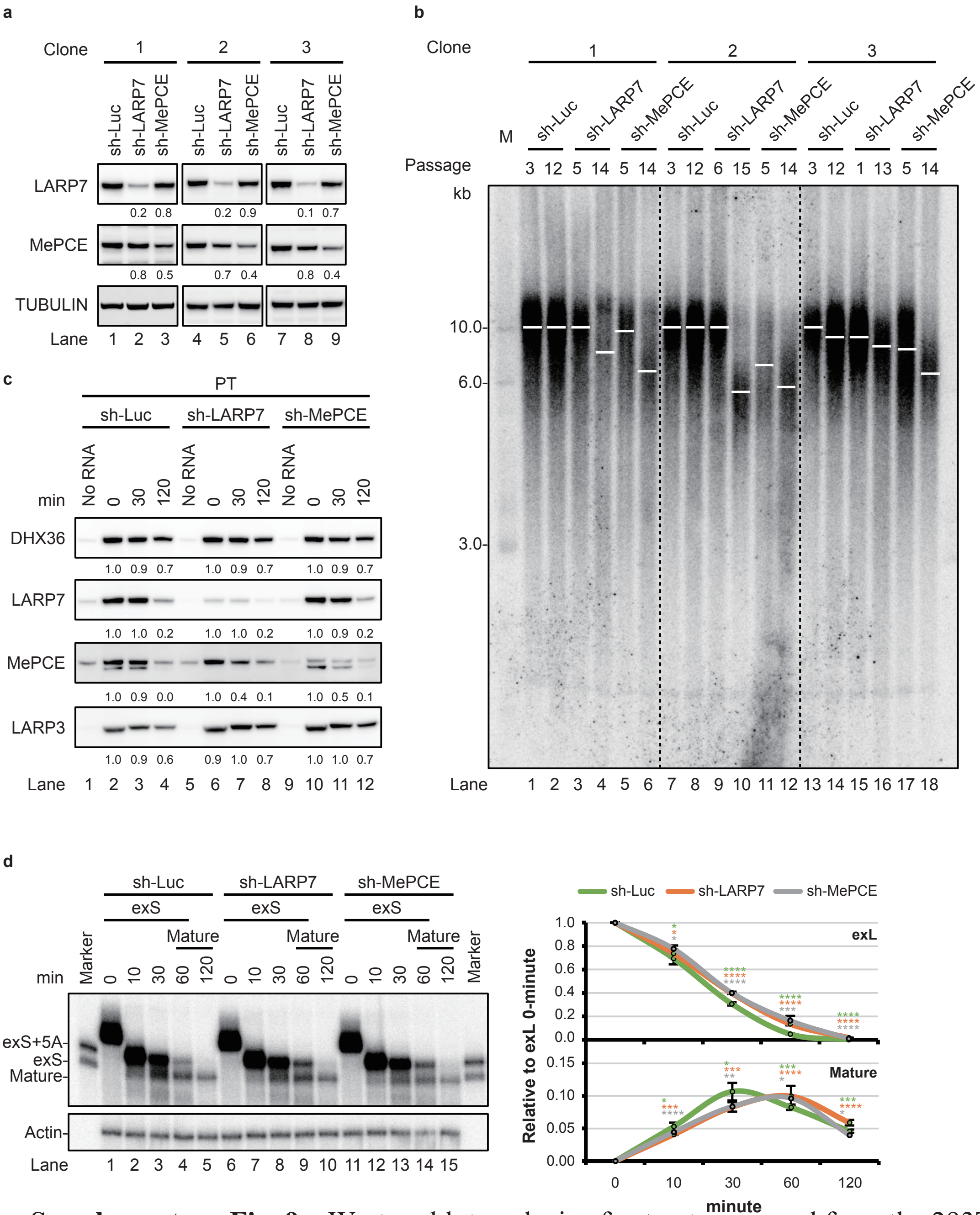

Supplementary Fig. 9 a Western blot analysis of extracts prepared from the 293T cells treated with shRNA targeting luciferase, LARP7, or MePCE. **b** Telomere lengths determined by TRF analysis of gDNA prepared from the 293T cells treated with shRNAs targeting luciferase, LARP7, or MePCE. **c** Western blotting analysis of telomerase assembled on biotin-labelled hTR in the indicated extracts, followed by pulldown with streptavidin beads for the indicated times. d The in vitro 3′ end processing assay with ^{32}P-labelled hTR fragments (from nucleotides 206 to 461 with an oligo A tail) was carried out in the indicated extracts at 37°C for the indicated times. RNA was purified and resolved on a 6% polyacrylamide gel containing 8 M urea. Actin served as the loading control.
