# Supplementary material for: LARP3, LARP7, and MePCE are involved in the early stage of human telomerase RNA biogenesis
Source: Nat Commun. 2024 Jul 15;15:5955. doi: 10.1038/s41467-024-50422-w (PMC11250828; doi:10.1038/s41467-024-50422-w)
Supplement: Supplementary file 4 — Source Data [file 41467_2024_50422_MOESM4_ESM.zip › SourceData/SourceData_Westernblots.pdf]

a

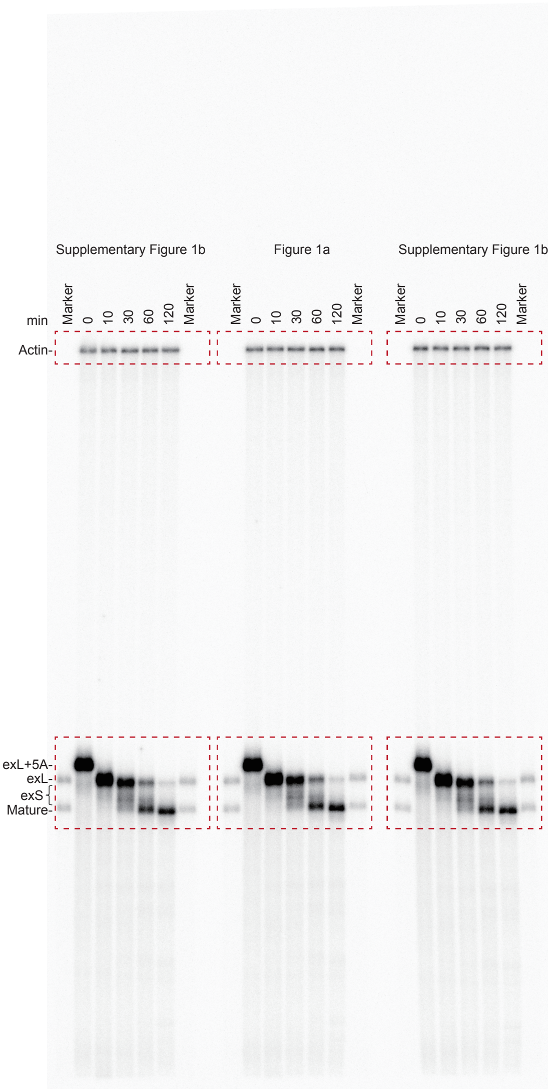

b

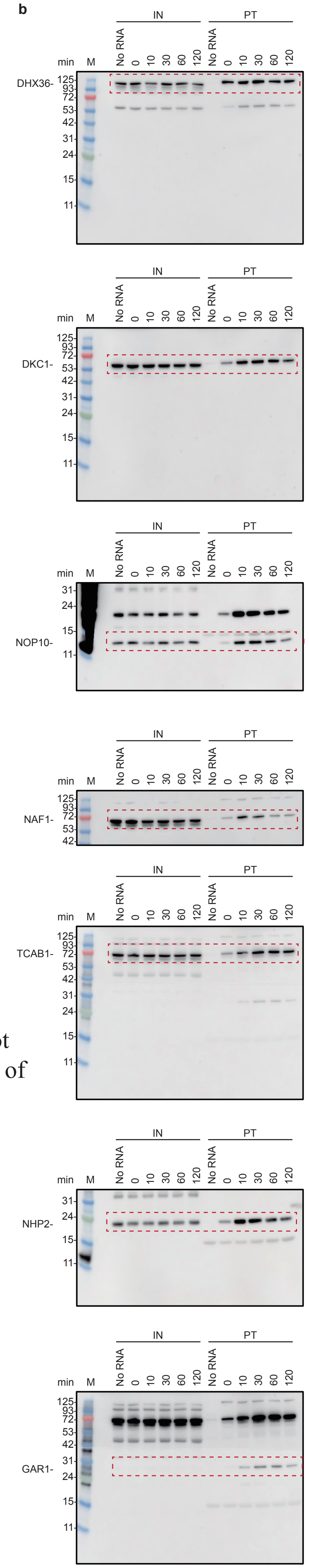

c

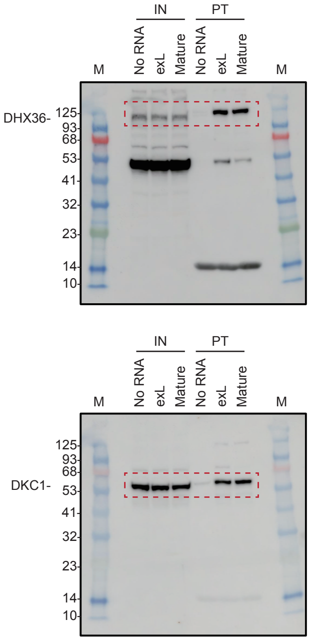

d

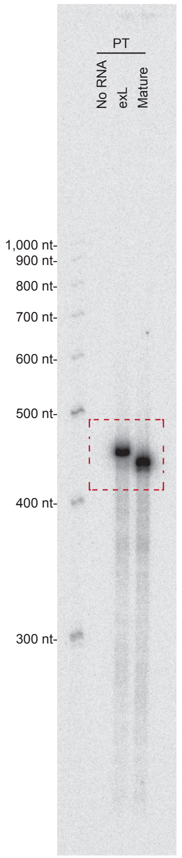

Source Data Full blots of Figure 1a and Supplementary Fig. 1b. **b** Full blots of Figure 1c. **c** Full Western blot images are shown in Figure 1d. **d** Full blots of the Northern blot shown in Figure 1d.

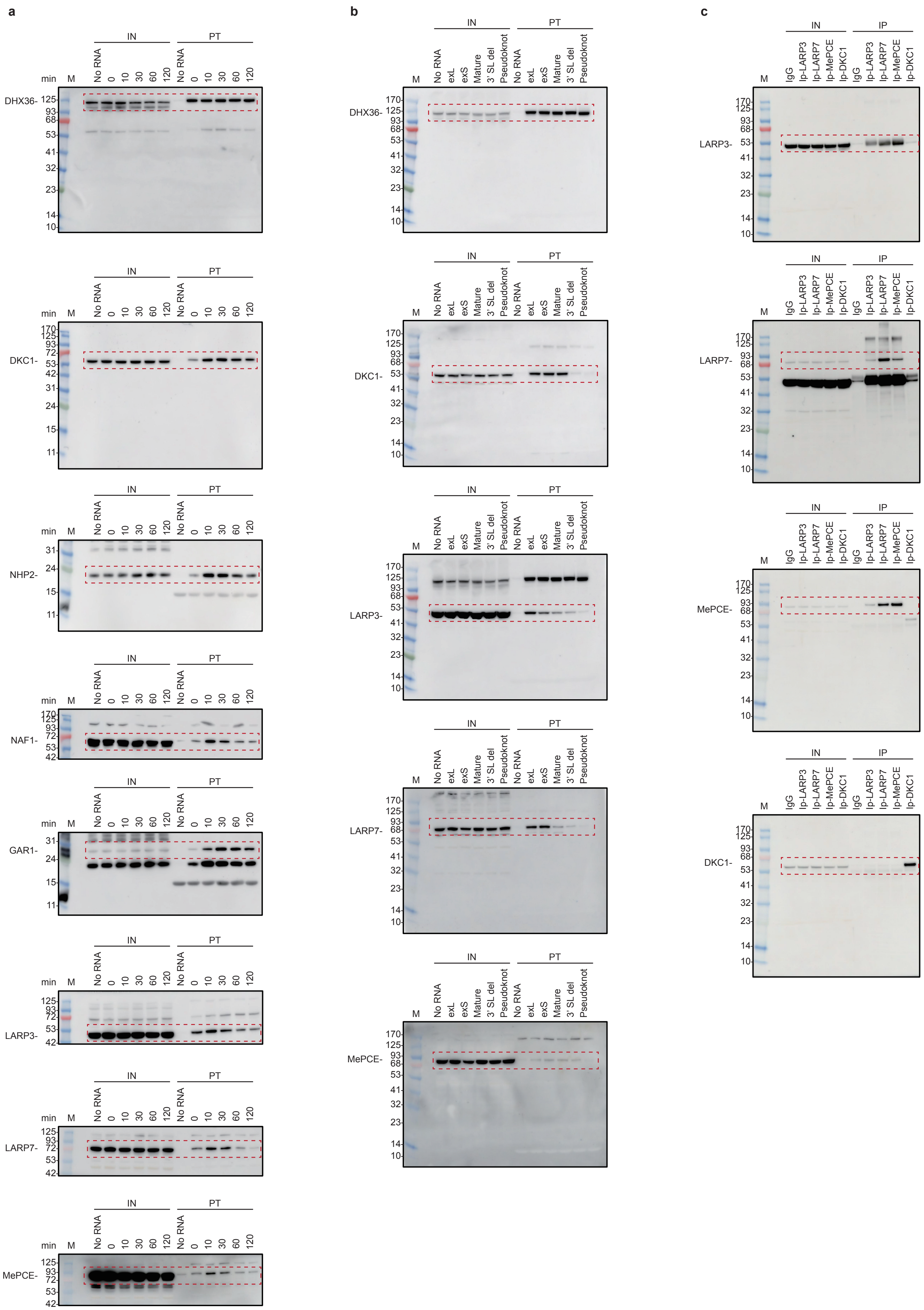

Source Data: Full blots of the data in Figure 2a. **b** Full blots of Figure 2b. **c** Full blots of Figure 2c.

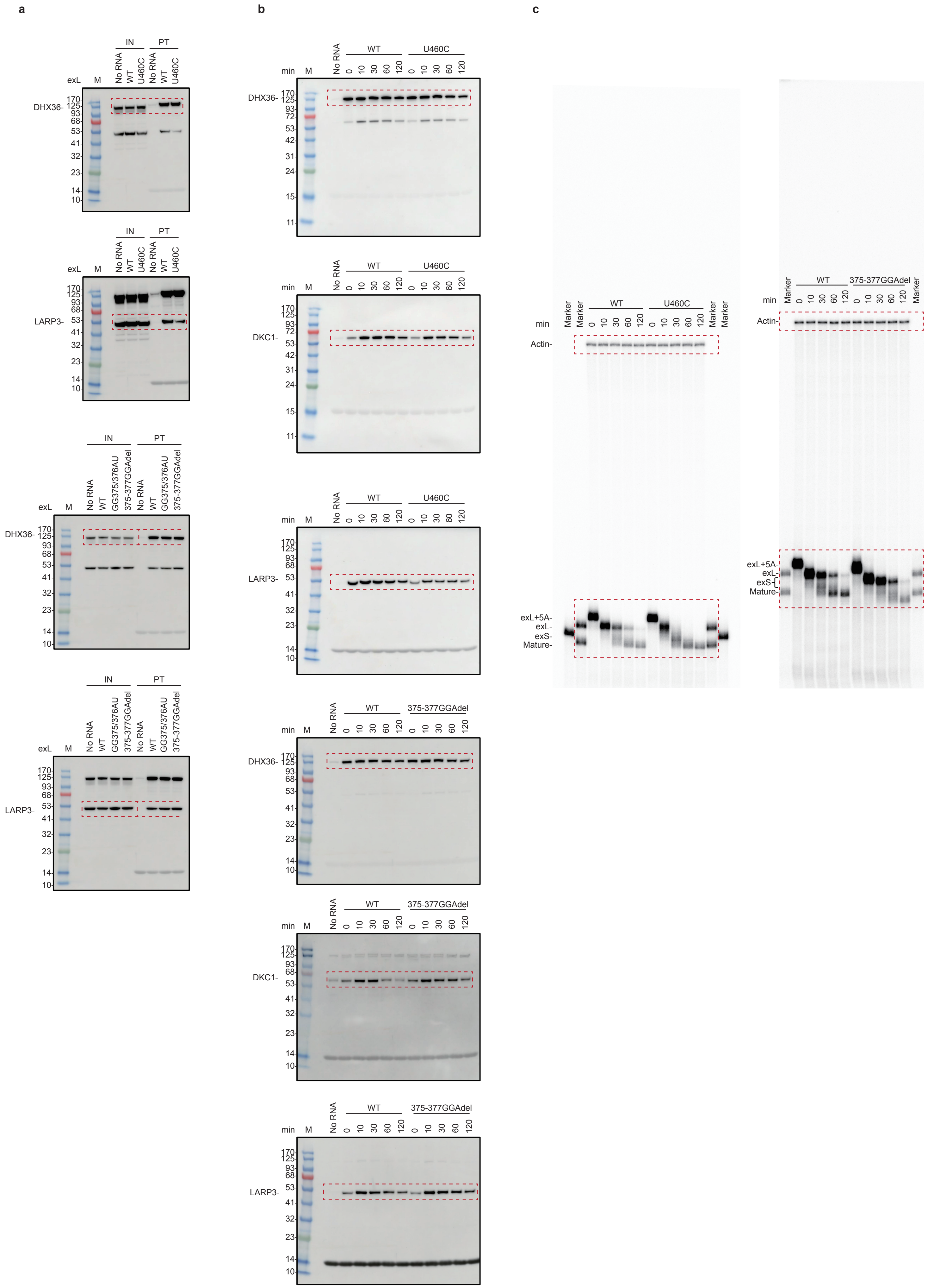

Source Data: Full blots of the data in Fig. 3b. **b** Full blots of Figure 3c. **c** Full blots of Figure 3d.

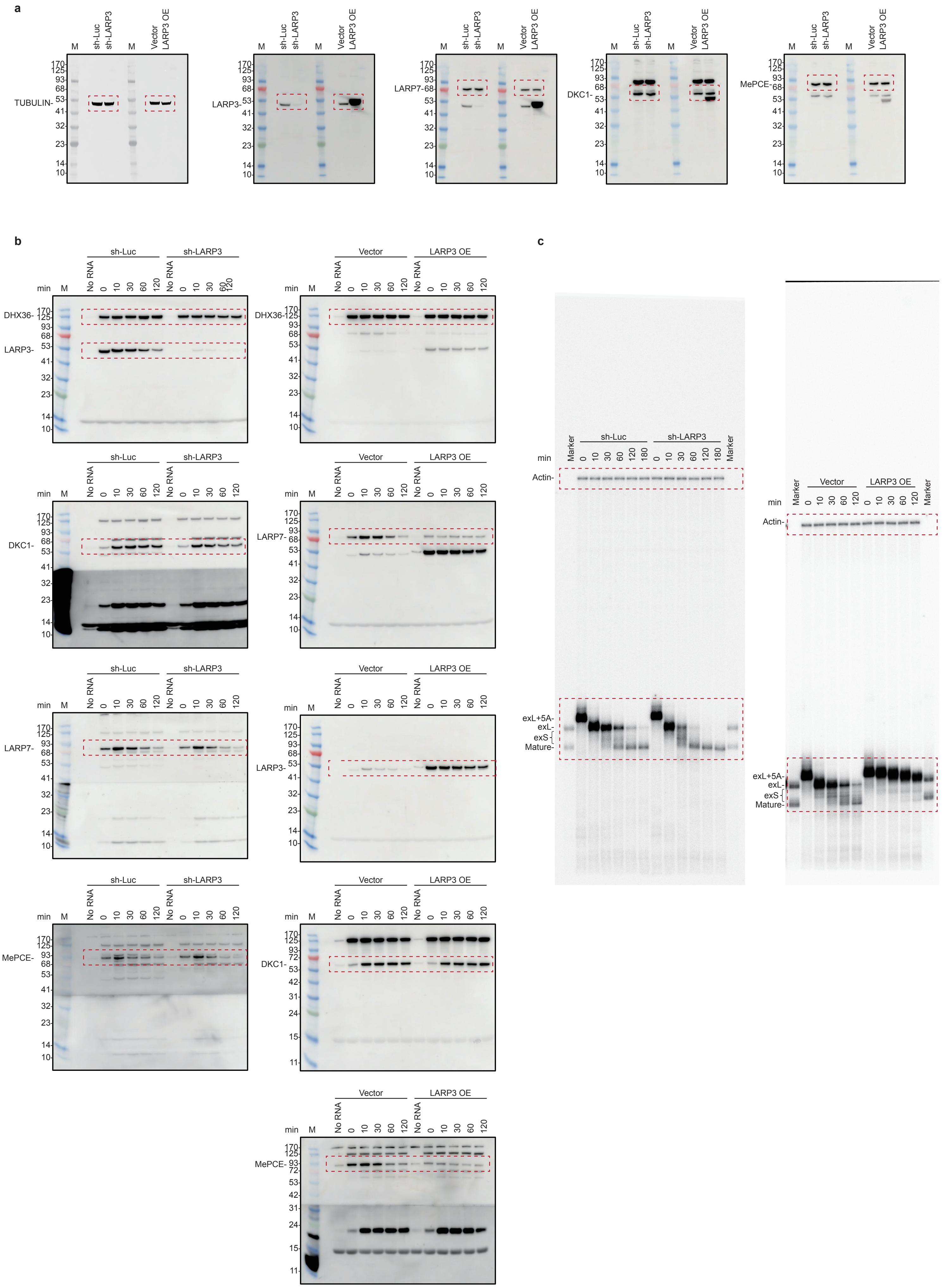

Source data Full blots of the data in Fig. 4a. **b** Full blots of Figure 4d. **c** Full blots of Figure 4f and 4g.

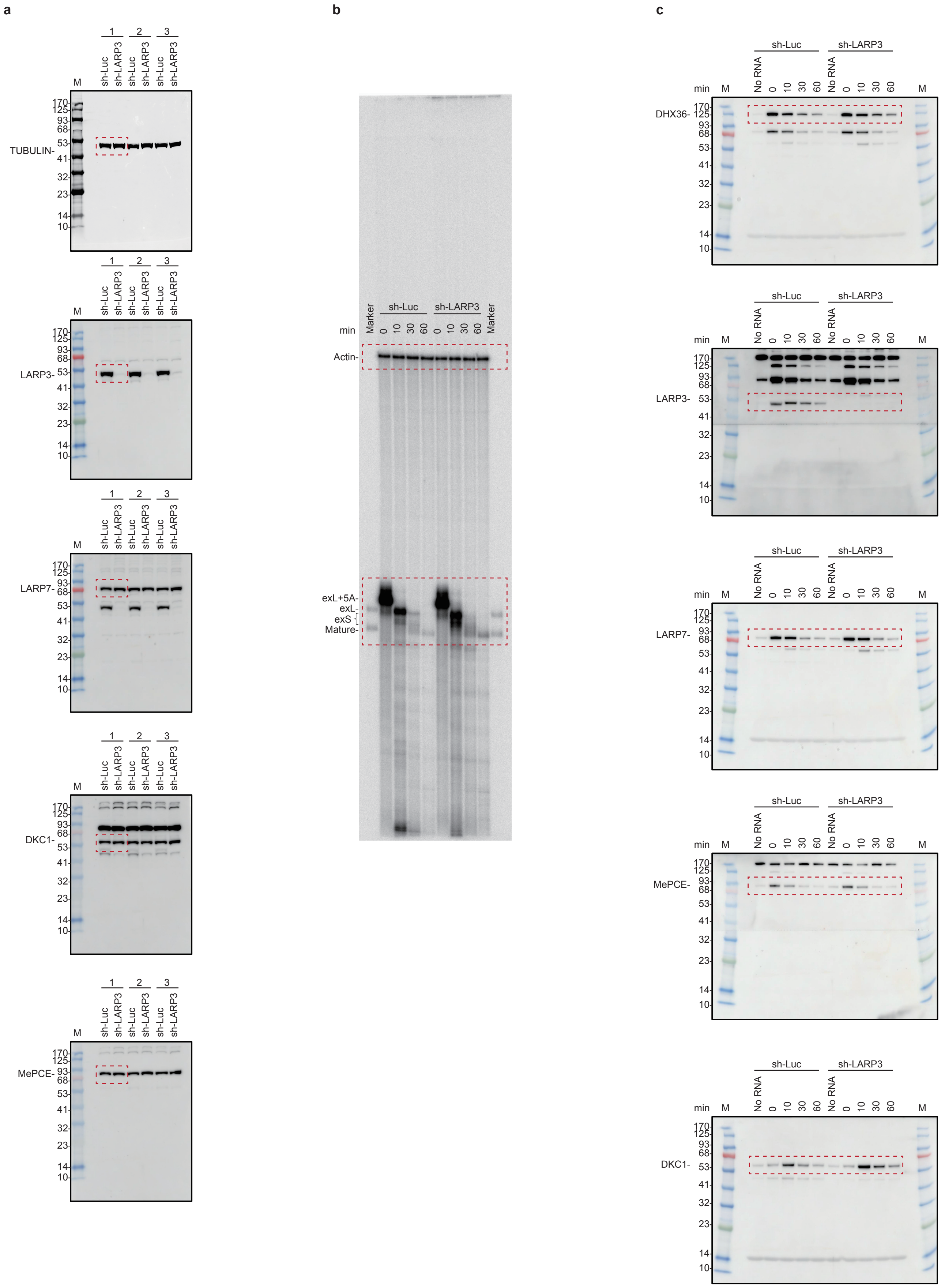

Source Data: Full blots of the data in Figure 5a. **b** Full blots of Figure 5c. **c** Full blots of Figure 5d.

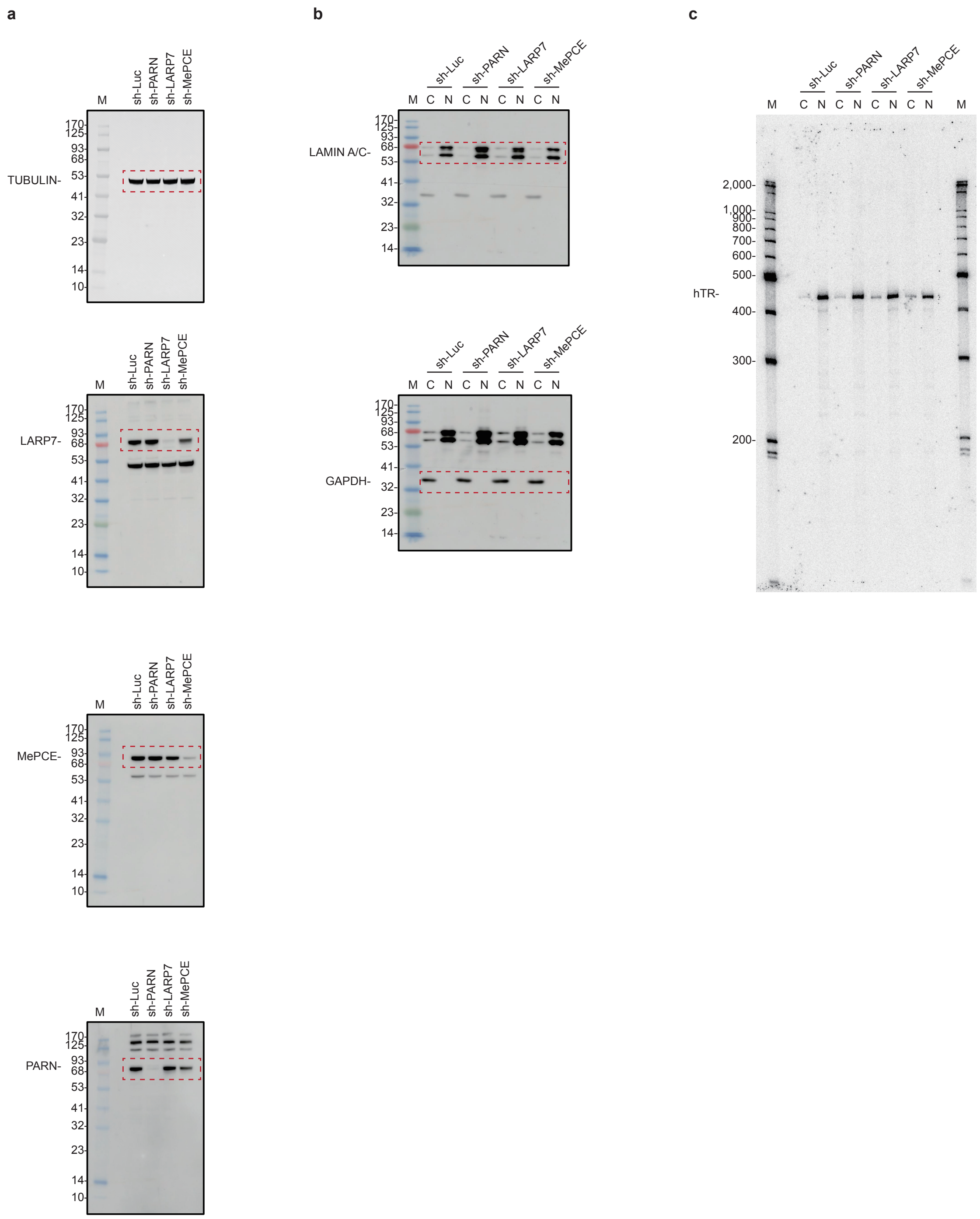

Source data: Full blots of the data in Fig. 6a. **b** Full Western blot images shown in Figure 6i. **c** Full blots of the Northern blot shown in Figure 6i.
